# Effects of High Gamma Doses on the Structural Stability of Metal–Organic Frameworks

**DOI:** 10.1021/acs.langmuir.2c01074

**Published:** 2022-07-11

**Authors:** Chao Ma, Huanhuan Liu, Hubert T. Wolterbeek, Antonia G. Denkova, Pablo Serra Crespo

**Affiliations:** Applied radiation and isotopes, Radiation Science and Technology, Faculty of applied sciences, Delft University of Technology, 2629 JB Delft, Mekelweg 15, The Netherlands

## Abstract

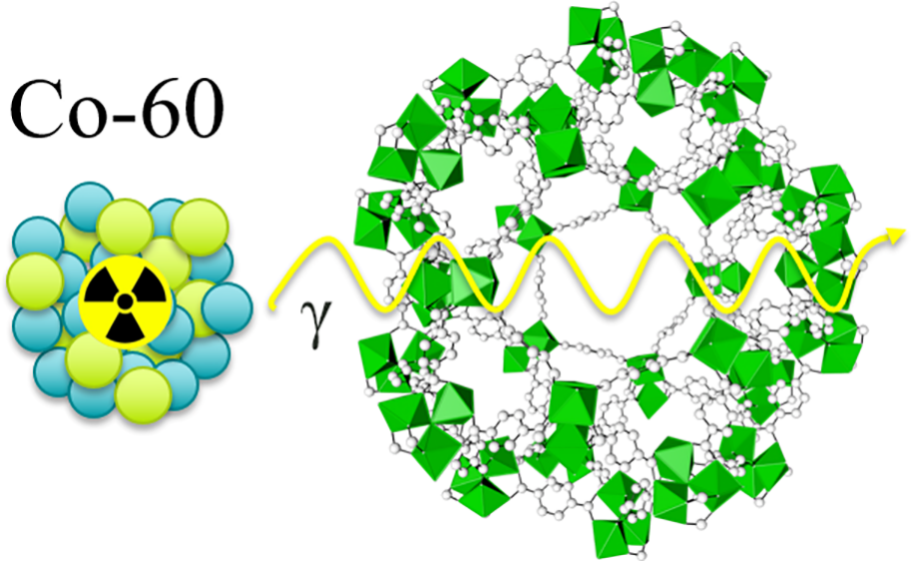

Four different MOFs were exposed to γ rays by a cobalt-60 source reaching a maximum dose of 5 MGy. The results showed that the MIL-100 (Cr) and MIL-100 (Fe) did not exhibit obvious structural damage, suggesting their excellent radiation stability. MIL-101 (Cr) showed good radiation stability up to 4 MGy, but its structure started degrading with increasing radiation dose. Furthermore, the results showed that the structure of AlFu MOFs started to decompose at a gamma dose of 1 MGy, exhibiting a much lower tolerance to γ radiation. At this radiation energy, the dominant interaction of the gamma-ray with MOFs is the Compton effect and the radiation stability of MOFs can be improved by prolific aromatic linkers, high linker connectivity, and good crystallinity. The results of this study indicate that MIL-100 and MIL-101 MOFs have a good potential to be employed in nuclear applications, where relatively high radiation doses play a role, for example, nuclear waste treatment and radionuclides production.

## Introduction

In recent years, increasing energy demand has renewed interest in nuclear energy, especially since it is an effective way to achieve carbon dioxide neutral energy production.^1^ Nevertheless, nuclear waste is still a major concern and has resulted in studies aiming at better waste management in which separation or sequestration of the different radionuclides is essential, especially in the long-run. On top of that, the production of radionuclides of medical interest is also a major topic in the nuclear field.^2^

Metal–organic frameworks (MOFs) materials have been investigated extensively as adsorbents for nuclear waste treatment as well as for radionuclide production, due to their high specific surface area, tunable functional groups allowing high selectivity, and good chemical stability.^3−6^ For instance, several MOFs have shown outstanding performance in the field of radioactive gas separation (^85^Kr, ^129^I, ^135^Xe, and ^222^Rn),^7−11^ seawater purification,^12−14^ radionuclide adsorption for wastewater remediation (^59^Fe, ^65^Zn, ^137^Cs, and ^235^U),^15−24^ and radionuclide production.^25^ Although these MOFs have excellent chemical stability and have shown great potential in waste treatment application, their resistance to ionizing radiation has hardly been reported. To fully exploit the potential of MOFs in these fields, it is imperative to determine their radiation resistance. So far, the stability of only a few MOFs has been investigated under γ radiation. For example, A series of SIFSIX-3 MOFs have been studied under beta and gamma irradiation by Elsadi et al., who reported that SIFSIX-3-Cu had the best radiation resistance to gamma and beta radiation up to a dose of 50 kGy and 25 MGy, respectively.^22^ The radiation stability of several MOFs with different metals (Al, Zr, Cu, Zn) have been studied under different gamma doses and the results have demonstrated that MIL-100 (Al) shows the best radiation tolerance, that is, 2 MGy.^23^ Gilson et al. have developed a thorium-based MOF, which has survived γ radiation up to a dose of 4 MGy and a dose of α particles up to 25.5 MGy.^26^ Furthermore, Nenoff et al. have investigated the influence of gamma dose rates (0.78 Gy/min, 3days and 423.3 Gy/min, 23 min) on the stability of NU-1000 and UiO-66, and found that NU-1000 exhibits better stability because of its high linker connectivity and lower node density.^27^ However, the radiation resistance of many other promising MOFs and especially at higher γ radiation dose remains unknown.

**Figure 1:**
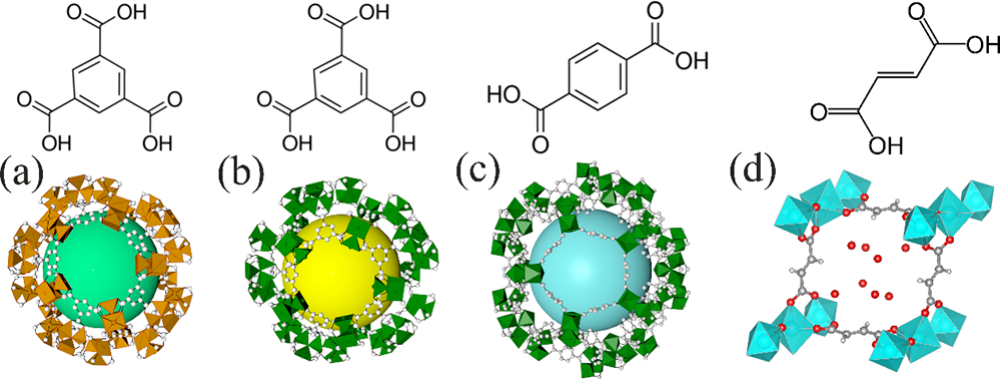
Illustration of the structure and corresponding organic linkers of (a) MIL-100 (Fe), (b) MIL-100 (Cr), (c) MIL-101 (Cr), and (d) AlFu MOFs. Iron, chromium, aluminum, carbon, and oxygen atoms are denoted in orange, green, blue, gray, and red colors, respectively.

MIL-101 (Cr), possessing high surface area and excellent chemical stability, has shown good potential to separate radionuclides.^28^ In addition, this MOF can also be applied to produce the ^51^Cr radionuclide, which is widely used to label platelets and red blood cells, and to evaluate their lifespan in clinical application and is desired radionuclide, requiring high specific activity (high activity per unit mass). Achieving high specific activity with nuclear reactors is hard but would be possible by the combination of Cr-based MOFs and hot atom approaches (Szilard-Chalmers effect).^29^ In order to realize the application of MIL-101 (Cr) in a highly radioactive environment, the effects of γ radiation on its structural evolution need to be explored. At the same time, it is very interesting to determine the influence of organic linkers and metal clusters on the radiation stability of MOFs in a more systematic fashion allowing rational choice of a MOF according to its application. Therefore, MIL-100 (Fe), MIL-100 (Cr), and aluminum fumarate MOFs (AlFu MOFs) have been studied and compared. The structure of these MOFs and corresponding organic linkers are shown in Figure 1. All MOFs in this study have been irradiated by gamma-ray from 0 Gy to 5 MGy, and their structural changes were monitored by XRD, SEM, FT-IR and nitrogen adsorption.

## Experimental Section

### Synthesis and Characterization

MIL-100 (Cr), MIL-100 (Fe), MIL-101 (Cr), and aluminum fumarate (AlFu) MOFs were synthesized according to previous literature.^30−33^ The details of the synthesis are shown in the Supporting Information (SI).

Powder X-ray diffraction patterns of the synthesized MOFs were obtained by a PANalytical X’Pert Pro pw3040/60 diffractometer with Cu Kα radiation operating at 45 kV and 40 mA. Brunauer–Emmett–Teller (BET) surface area of the samples was collected on a Micromeritics Tristar II at 77 K, and all samples were pretreated at 200 C for 15 h before measurement. Fourier transform infrared spectra (FTIR) of powder samples was directly measured by a PerkinElmer Spotlight 400 FT-IR spectrometer with a range of 650–2500 cm^–1^. The morphology and particle size of the samples were determined by scanning electron microscopy (SEM, JEOL, JSM-IT100). X-ray photoelectron spectra (XPS) was collected using a ThermoFisher Al K-alpha apparatus and scans were performed by a 400 μm spot size with an energy step size of 0.2 eV. The thermal stability of four MOFs was investigated by thermogravimetric analysis (TGA) using a Mettler-Toledo/STDA 851e apparatus with a heating rate of 5 C/min.

### Gamma Irradiation

The gamma source (GC220) is an irradiation cell using the radionuclide ^60^Co (as shown in SI Figure S1), which was used to study the effects of gamma irradiation on the selected MOFs. The cobalt-60 (1.17, 1.33 MeV) source has a half-life of 5.272 years. We packed ∼0.2 g of powder of each sample in Posthumus plastic capsules in air and irradiated for different times to achieve different doses. The gamma dose was calculated according to eq 1-1:
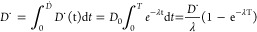
1Where *D* and *Ḋ Ḋ* are dose and dose rate, respectively. The initial dose rate was 0.65 kGy/h and λ (3.6 × 10–4 d^–1^) is the decay constant of ^60^Co. The radiation time (*T*) was 32.8 days (0.5 MGy), 66 days (1.0 MGy), 99 days (1.5 MGy), 133 days (2.0 MGy), 199 days (3.0 MGy), 269 days (4.0 MGy), and 340.5 days (5.0 MGy), respectively.

## Results and Discussion

To evaluate the radiation stability, the structure changes or the loss of crystallinity of the synthesized MOFs were monitored through XRD, SEM, FT-IR, and nitrogen adsorption after exposure to a cobalt-60 source at different doses of γ radiation.

**Figure 2:**
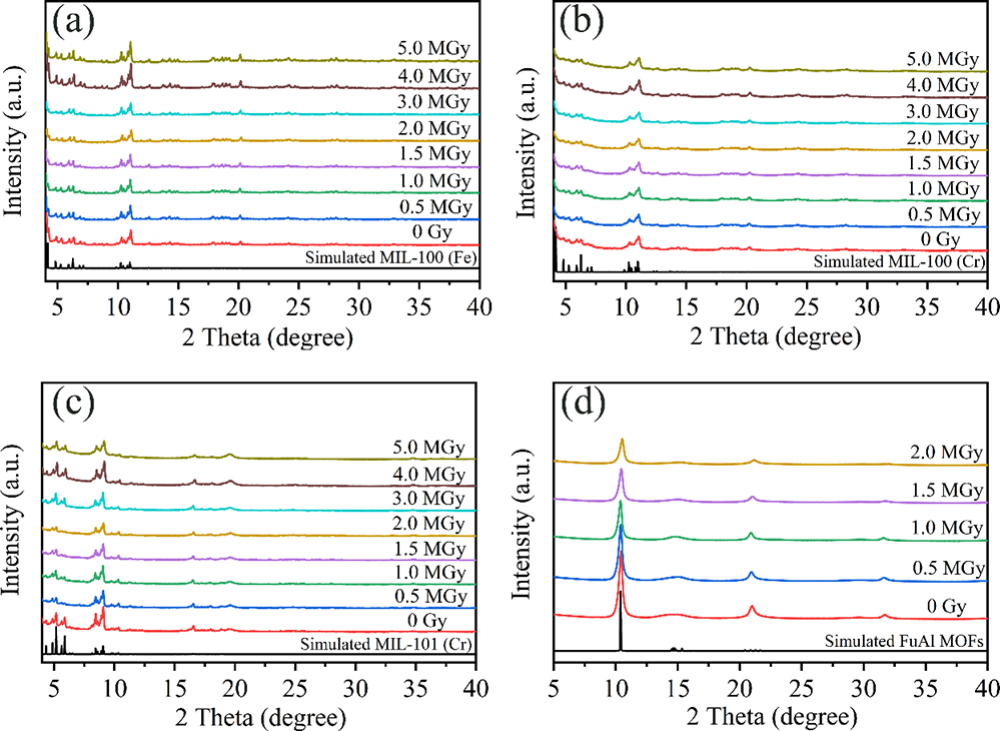
XRD patterns of (a) MIL-100 (Fe), (b) MIL-100 (Cr), (c) MIL-101 (Cr), and (d) FuAl MOFs exposed to different gamma doses.

Figure 2 shows the XRD patterns of MIL-100 (Fe), MIL-100 (Cr), MIL-101 (Cr), and AlFu MOFs before and after the irradiation at different γ radiation doses. Their diffraction peaks are consistent with the simulated patterns for each material. After exposure to different γ radiation doses, the diffraction peaks of MIL-100 (Fe) (Figure 2(a)), MIL-100 (Cr) (Figure 2(b)) and MIL-101 (Cr) (Figure 2(c)) maintained the same diffraction patterns. The full-width half-maximum (fwhm) of the most intense peak in XRD patterns was analyzed and shown in SI Figure S2. There is a small variation in the values of fwhm for MIL-100 (Fe) and MIL-100 (Cr). The values of fwhm for MIL-101 (Cr) increased slightly at relatively low radiation dose (from 0 to 4 MGy) and the value increased significantly (22%) when exposed to 5 MGy, showing severe loss of crystallinity. The diffraction peaks of AlFu MOF became broader as the gamma dose increased (Figure 2(d)). When the γ radiation dose was lower than 1 MGy, the fwhm of AlFu MOF remained stable. The value of fwhm increased from 0.4 to 0.51 after receiving 2 MGy of gamma irradiation, resulting in broader diffraction peaks.

**Figure 3:**
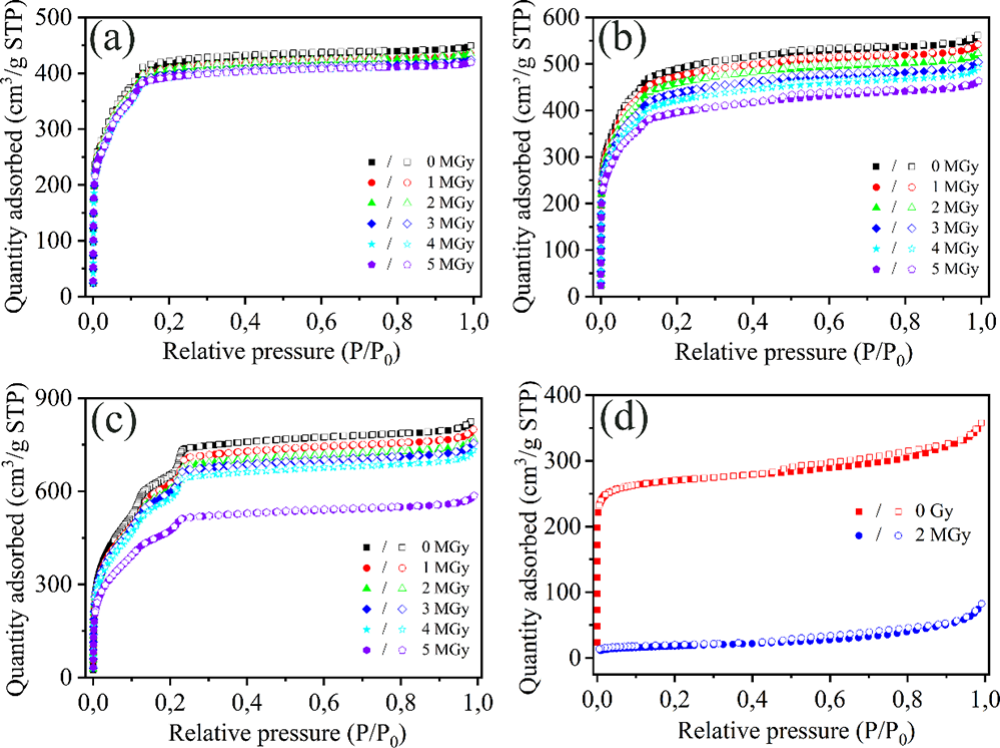
N_2_ adsorption isotherms of (a) MIL-100 (Fe), (b) MIL-100 (Cr), (c) MIL-101 (Cr), and (d) AlFu MOFs exposed to different gamma doses.

The surface area of the four materials was determined by N_2_ adsorption at 77 K. Figure 3(a) shows the N_2_ adsorption–desorption isotherms of MIL-100 (Fe) after exposure to the different gamma doses. MIL-100 (Fe) possessed type I adsorption isotherm and had a surface area of 1574 m^2^/g. After exposure of 1 and 2 MGy, its surface area was 1527 and 1528 m^2^/g (having a 3% drop), respectively. When MIL-100 (Fe) was exposed to higher irradiation doses (between 3 and 5 MGy) its surface area decreased to 1513 m^2^/g, 1498 m^2^/g, and 1507 m^2^/g (as shown in SI Table S1).

The SEM images in SI Figure S3 show that MIL-100 (Fe) exhibited a rod-like shape with inhomogeneous size. There were no detectable changes observed to the particle morphology and size after the different radiation exposures. The FT-IR spectrum of MIL-100 (Fe) can be found in SI Figure S4. The spectrum displays two peaks at 1625 and 1382 cm^–1^ that are attributed to asymmetric and symmetric vibrations of carboxyl groups,^34^ respectively. The peak at around 1445 cm^–1^ is assigned to stretching vibrations of the O–C–O group.^35^ No obvious changes can be observed in the FT-IR spectra, which is consistent with the XRD analysis.

Figure 3(b) shows the N_2_ adsorption isotherms of MIL-100 (Cr) after different gamma irradiation doses. The BET surface area of MIL-100 (Cr) calculated from nitrogen adsorption isotherm was 1862 m^2^/g before gamma irradiation. The shape of the adsorption isotherms was the same as that of fresh MIL-100 (Cr), but they were found to decrease gradually in adsorption capacity. Correspondingly, their surface area decreased by 3.2% (1802 m^2^/g), 6.3% (1744 m^2^/g,) 10.8% (1660 m^2^/g), 14.1% (1600 m^2^/g), and 18.9% (1510 m^2^/g) after receiving gamma doses of 1 MGy, 2 MGy, 3 MGy, 4 MGy and 5 MGy, respectively, where there was an approximately 4% decrease in surface area for each gamma dose. SEM images (SI Figure S5) showed that the morphology of MIL-100 (Cr) was unchanged. But the crystal size decreased a little after high γ radiation exposure (4 MGy and 5 MGy). The FT-IR spectrum of MIL-100 (Cr) was measured (as shown in SI Figure S6) and no obvious changes could be observed with γ radiation up to 5 MGy.

Nitrogen adsorption isotherms of MIL-101 (Cr) after exposure to the different gamma doses are shown in Figure 3(c). The BET surface area of the fresh sample was 2203 m^2^/g. After receiving gamma doses of 1 MGy, 2 MGy, 3 MGy, and 4 MGy, the BET surface area of MIL-101 (Cr) decreased from 2203 m^2^/g to 2159 m^2^/g, 2101 m^2^/g, 2072 m^2^/g, and 2099 m^2^/g, respectively (SI Table S1). The BET surface area of MIL-101 (Cr) was reduced by only 4.7% when exposed to a gamma dose of 4 MGy. When the gamma dose reached 5 MGy, MIL-101 (Cr) decreased by 20.0% in surface area (1762 m^2^/g). Its micropore volume decreased from 1.0715 cm^3^/g to 0.7508 cm^3^/g (30% reduction, as shown in SI Table S2). Additionally, the microstructure and morphology of MIL-101 (Cr) particles were examined by SEM, as shown in SI Figure S7. The images show that the MIL-101 (Cr) particles are irregular spheres with a relatively uniform distribution. The morphology and size of the particles did not show any obvious changes after exposure to different gamma doses.

Figure 3(d) shows the N_2_ adsorption isotherms of AlFu MOFs. The surface area of AlFu MOFs at 2 MGy had a significant decrease (63 m^2^/g) when compared to the original surface area of 1070 m^2^/g. SEM images in SI Figure S9 showed that the morphology of MIL-100 (Cr) was unchanged. The damage to the structure of AlFu MOFs was further confirmed by FT-IR spectra (see SI Figure S10). The C=C vibrations at 1600 cm^–1^ and O–H bending vibrations of the aluminum clusters^36,37^ at 998 cm^–1^ could not be observed after 1 MGy radiation.

**Figure 4:**
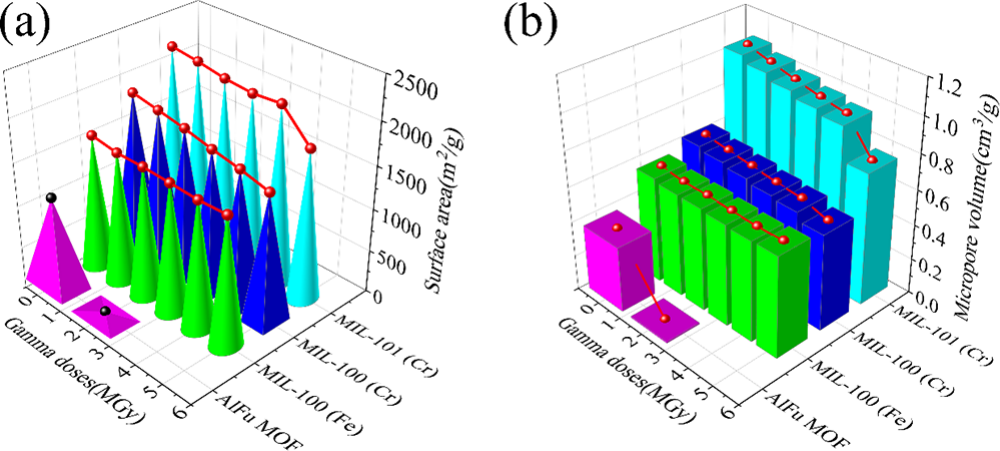
3D representations of the four MOFs (a) surface area and (b) micropore volume as a function of the γ radiation dose. Red lines represent the observed trend with increasing gamma dose.

The radiation stabilities of four MOFs were explored by determining their most important characteristics (e.g., crystallinity). The small fluctuation value of fwhm and the lack of significant reduction in BET surface area (Figure 4a) after receiving γ radiation of 5 MGy indicate that MIL-100 (Fe) has excellent radiation stability toward γ radiation. After exposure to a γ radiation dose of 5MGy, the XRD pattern and fwhm of MIL-100 (Cr) did not have obvious changes, suggesting that it kept good crystallinity. Although a small decrease in surface area and micropore volume (Figure 4b) could be observed with increasing gamma doses, MIL-100 (Cr) showed tenacious resistance to gamma irradiation, exhibiting as good radiation stability as MIL-100 (Fe). The TGA curves also demonstrated that they still kept good thermal stability after receiving 5 MGy of gamma irradiation (see SI Figure S11). Furthermore, MIL-101 (Cr) showed the same performance as MIL-100 (Cr) when it was irradiated by γ rays at doses of 1, 2, 3, and 4 MGy. Its surface area and micropore volume had a slight decrease, but it also demonstrated that this MOF is highly resistant up to 4 MGy of a gamma dose. Subsequently, its surface area decreased significantly (20%) and the fwhm value also increased visibly, suggesting that the structure of MIL-101 (Cr) started decomposing at a gamma dose of 5 MGy, causing lower decomposing temperature (see the SI Figure S11(c)). In addition, the crystallinity of AlFu MOF began to degenerate after receiving 1 MGy of γ radiation and its pore structure characteristics completely disappeared at a gamma dose of 2 MGy, exhibiting much lower radiation stability, which could also be proved by the TGA curve (SI Figure S11(d)). Why the different MOFs react differently when exposed to γ radiation is not clear but there seem to be certain clues that can explain why one material is more stable than another.

**Figure 5:**
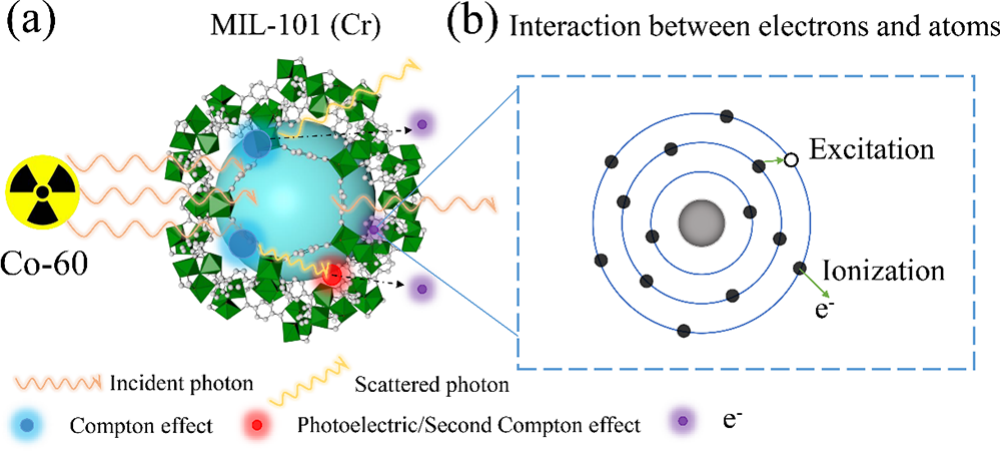
Schematic illustration (a) of interactions between MIL-101 (Cr) with gamma-rays (b) interaction of traveled electrons with atoms in framework of MOFs.

Three main processes rule the interaction of gamma-ray with matter, namely the photoelectric effect, the Compton effect, and the pair production. The probability of these interactions strongly depends on the atomic number (Z) and the energy of gamma-ray.^38^ The Compton effect is expected to be the most dominant based on the gamma energy (*E*γ = 1.17, 1.33 MeV) and the *Z* of the elements comprising the MOFs. The Compton process consists in a partial transfer of energy to an electron in the MOF resulting in the energy loss of the γ radiation, which can then further interact with other electrons, accompanied by the second Compton effect or photoelectric effect, and thus generating recoil electrons (Figure 5(a)). Some of the Compton electrons could travel to air without collision and the other electrons can collide with the orbital electrons of surrounding atoms in the MOFs by incoherent scattering, causing ionizations or excitations (Figure 5(b)). Finally, the energy of the excited atoms can be dissipated through the emission of fluorescence or Auger electrons, and, in the last instance, through vibration (heat dissipation).

As discussed above, the structure of the MOF seems to affect its stability toward radiation. First, the mass-energy absorption coefficient is an effective index to measure the average fraction of photon energy absorbed by materials. Metal clusters of MOFs in this research consist of metal and oxygen atoms, which have a much higher total attenuation coefficient than organic linkers, suggesting that metal clusters can act as radiation antennas.

As shown in Table 1, Al metal atom had the lowest photon cross-section, but AlFu MOF had the worse radiation stability compared with the other three MOFs, which could be attributed to the lack of aromaticity of the organic linkers. The aromatic linkers can promote delocalization and migration of excitations based on high energy delocalization.^39,40^ Therefore, the aromaticity of the linker has a significant impact on the radiation stability of MOFs under gamma-ray environment. Second, MIL-100 (Fe) and MIL-100 (Cr), which have the same linker and crystal structure, showed good irradiation stability. The surface area of MIL-100 (Cr) reduced a little bit more (16%), although Cr has a lower photon cross-section. The larger fwhm value of MIL-100 (Cr) indicated that it had a worse crystallinity compared with MIL-100 (Fe). The formed defects could be not beneficial for the energy transfer and dissipation,^41^ resulting in low tolerance ability for γ radiation, which could be the reason for the better stability of MIL-100 (Fe).

**Table 1:** Total Photon Cross Sections (Barns) Of Different Metals, 1 Barn = 10^–24^ cm^2^

metal	energy (MeV)	total attenuation (barns/atom)
Al	1.17	2.55
	1.33	2.39
Cr	1.17	4.74
	1.33	4.44
Fe	1.17	5.51
	1.33	4.82
C	1.17	1.17
	1.33	1.09
O	1.17	1.56
	1.33	1.46
H	1.17	0.19
	1.33	0.18

**Figure 6:**
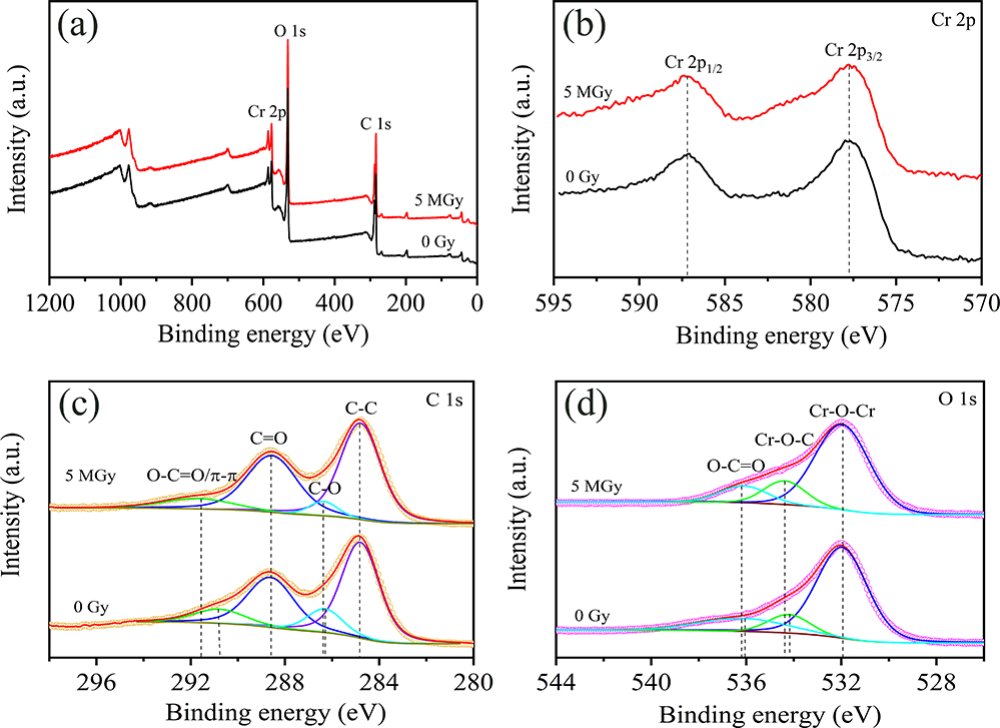
XPS spectra of MIL-100 (Cr) before and after (5 MGy) gamma irradiation; (a) XPS survey, (b) Cr 2p, (c) C 1s, and (d) O 1s.

Figure 6 shows the XPS spectra of MIL-100 (Cr) before and after a γ radiation dose of 5 MGy. It can be seen that the sample contains Cr, O, and C elements according to the XPS survey (Figure 6a). The Cr 2p spectrum is shown in Figure 6(b) and two peaks at 577.6 and 587.2 eV are ascribed to Cr 2p_3/2_ and Cr 2p_1/2_, respectively.^42^ The binding energy shifts cannot be observed suggesting that the metal clusters in MIL-100 (Cr) maintain integrity after exposure to a gamma dose of 5 MGy. The C 1s XPS (Figure 6(c)) spectrum of MIL-100 (Cr) before irradiation could be deconvoluted into four peaks at binding energies of 284.8 eV, 286.2 eV, 288.5 eV, and 290.6 eV, which were attributed to C–C/C–H, C–O, C=O, and O—C=O/π–π, respectively.^43^ Apparently, the peaks ascribed to O—C=O/π–π and C–O positively shift to the binding energy of 291.6 and 286.3 eV, implying the decrease of electron density of the carboxyl groups.^44^ The O 1s spectrum of MIL-100 (Cr) (Figure 6(d)) could be deconvoluted into three peaks at 531.9 eV, 534.2 eV, and 536.1 eV, which were ascribed to Cr–O–Cr, Cr–O–,C and O—C=O, respectively.^45^ After gamma irradiation, the two peaks of Cr–O–C and O—C=O shift to higher binding energies by 0.2 and 0.1 eV, which were attributed to decreased electron density of the junction of metal clusters and organic linkers, indicating that the bonds between Cr–O clusters and the carboxylate of H_3_BTC linkers could be partly broken during irradiation, resulting in the formation of defects. Third, MIL-100 (Cr) and MIL-101 (Cr), which have the same metal clusters but different connecting linkers, had good radiation stability under γ radiation of 4 MGy. However, MIL-101 (Cr) started to decompose with increasing gamma dose, which is related to the linker connectivity. Since each linker in MIL-100 (Cr) is connected with three metal clusters and the structure can still be kept when one or two connection sites are broken, causing this material to exhibit better stability. Another possible explanation could be that the metal nodes concentration per volume unit of MIL-100 (Cr) is slightly higher than that of MIL-101 (Cr), indicating that closer metallic atoms might attenuate more the generated electrons, which is instrumental in stabilizing the structure of MOFs.

To better understand the relationship between structural characteristics and radiation stability of the MOFs, more systematic experiments need to be carried out to explore other possible factors that determine the stability of MOFs. In addition, more studies should be carried out to determine the maximum radiation dose that the MOFs can tolerate without loss of their characteristic properties.

## Conclusions

In conclusion, MIL-100 (Fe), MIL-100 (Cr), MIL-101 (Cr), and AlFu MOFs were prepared and irradiated using γ rays at doses ranging from 0 Gy to 5 MGy. The structure of all materials was characterized by XRD, BET, SEM, and FT-IR. The MIL-100 (Fe) and MIL-100 (Cr) presented outstanding stability when exposed to radiation of high doses (5 MGy). MIL-101 (Cr) exhibited good radiation stability when the material was subjected to gamma doses within a range of 0–4 MGy. A sudden decrease in the surface area demonstrated that MIL-101 (Cr) started to be damaged with increasing gamma dose. Meanwhile, the XRD results of AlFu MOF proved that the crystallinity of AlFu MOFs suffered a severe loss after receiving 1 MGy gamma dose. The BET and FI-IR results indicated that the structure of AlFu MOFs collapsed after exposure to high radiation dose. According to the structural analysis, the linker aromaticity plays an important role in the radiation stability of the MOFs. Additionally, high linker connectivity and good crystallinity of MOFs can also strengthen their radiation stability. To fully examine the potential of MOFs in nuclear applications their resistance to even higher doses should be assessed in the future.

## Supporting Information Available

Detailed information on materials, synthetic procedures, Cobalt-60 irradiation setup, SEM images, FT-IR spectra, TGA curves, (Tables S1 and S2) summary of the BET surface area and micropore volume (PDF)
