# Supplementary material for: Effects of High Gamma Doses on the Structural Stability of Metal–Organic Frameworks
Source: Langmuir. 2022 Jul 11;38(29):8928–33. doi: 10.1021/acs.langmuir.2c01074 (PMC9330767; doi:10.1021/acs.langmuir.2c01074)
Supplement: Supplementary file 1 — la2c01074_si_001.pdf [file la2c01074_si_001.pdf]

Supporting Information

Effects of high gamma doses on the structural stability of metal-organic frameworks

Chao Ma,[1] Huanhuan Liu,[1] Hubert T. Wolterbeek,[1] Antonia G. Denkova,[1,*] Pablo Serra Crespo[1,*]

[1]Applied radiation and isotopes, Radiation Science and Technology, Faculty of applied sciences, Delft University of Technology, 2629 JB Delft, Mekelweg 15, The Netherlands.

[*]Corresponding authors:

Antonia G. Denkova

E-mail: A.G.Denkova@tudelft.nl

Pablo Serra Crespo

E-mail: P.SerraCrespo@tudelft.nl

Table of Contents

Synthesis **S3**

Supplementary Figures and Table **S5**

Figure S1 .... **S5**

Figure S2 .... **S5**

Figure S3 .... **S6**

Figure S4 .... **S6**

Figure S5 .... **S7**

Figure S6 .... **S7**

Figure S7 .... **S8**

Figure S8 .... **S8**

Figure S9 .... **S9**

Figure S10 .... **S9**

Figure S11 .... **S10**

Table S1 .... **S11**

Table S2 .... **S11**

S 1. Synthesis

1.1 Material

1,3,5-benzenetricarboxylic acid (H_3BTC, 95%), 1,4-benzene dicarboxylic acid (H_2BDC, 98%) and sodium hydroxide (NaOH, ≥ 98%) were purchased from Sigma-Aldrich. Chromium chloride hexahydrate ($CrCl_3·6H_2O$, ≥ 96%), chromium nitrate nonahydrate ($Cr(NO_3)_3·9H_2O$, ≥ 98%), fumaric acid (>98%) and aluminum sulfate octahydrate ($Al_2(SO_4)_3·18H_2O$, 98%) were provided by Merck. Ferric nitrate nonahydrate ($Fe(NO_3)_3·9H_2O$, ≥ 99%) was provided by Fluka. All chemical reagents were used without any purification.

1.2 Synthesis of MIL-100 (Cr)

1.05 g of $CrCl_3·6H_2O$ and 0.42 g H_3BTC were mixed in a mortar and grounded using hand for 1 h at room temperature. Then the powder was transferred to a Teflon lined-autoclave and heated at 220 °C for 15 h in an electric oven. The obtained powder was washed with deionized water at 70 °C and ethanol at 60 °C for 3 h, respectively. The final collected product was dried at 150 °C for 12 h under vacuum.

1.3 Synthesis of MIL-101 (Cr)

Typically, 1.6 g of $Cr(NO_3)_3·9H_2O$ and 664 mg of H_2BDC were added into 19.2 ml deionized water. Then 0.177 ml of HNO_3 was added to the mixture, which was further sonicated to obtain a homogeneous solution. Finally, the solution was transferred to a Teflon lined-autoclave and heated at 220 °C for 8 h. To remove the unreacted reagents, the obtained precipitation was washed with DMF at 65 °C for 2 h and EtOH at 80 °C

for 24 h. Then the powder was washed with hot water at 80 °C for 5 h and finally dried at 120 °C overnight.

1.4 Synthesis of MIL-100 (Fe)

3.03 g of $Fe(NO_3)_3 \cdot 9H_2O$ and 1050.7 mg of H_3BTC were mixed and grounded by hand for half an hour. The mixture was transferred to a Teflon lined-autoclave and heated at 160 °C for 4 h. The collected product was washed with deionized water and ethanol for three times, respectively. The final sample was dried at 150 °C for 12 h under vacuum.

1.5 Synthesis of AlFu MOF

Aluminum fumarate MOF was synthesized using the hydrothermal method. 14.728 g of $Al_2(SO_4)_3 \cdot 18H_2O$ was dissolved in 70 ml of deionized water and the solution was heated at 60 °C for one hour. Another solution containing NaOH (5.32 g) and fumaric acid (5.104 g) was added to 70 ml of water and heated under 60 °C for one hour, which was then pumped to the above solution within 15 min. The suspension was kept at 60 °C for 30 min. the final product was washed with deionized water and ethanol for three times, respectively. The sample was dried at 80 °C for 12 h.

Supplementary Figures and Table

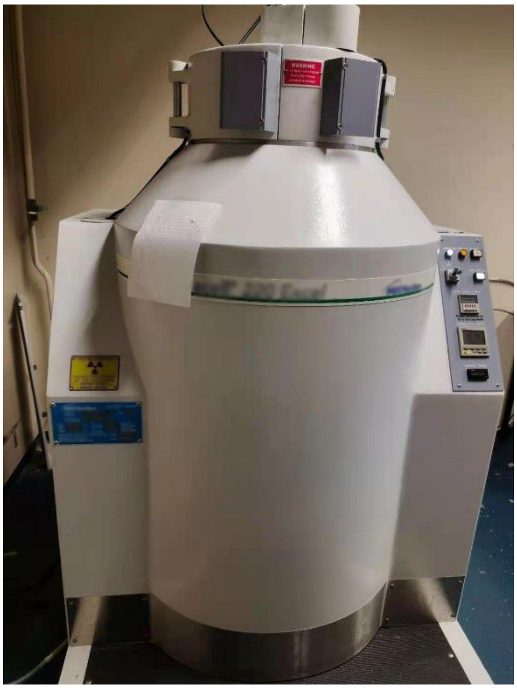

Figure S1. The facility of Cobalt-60 sources.

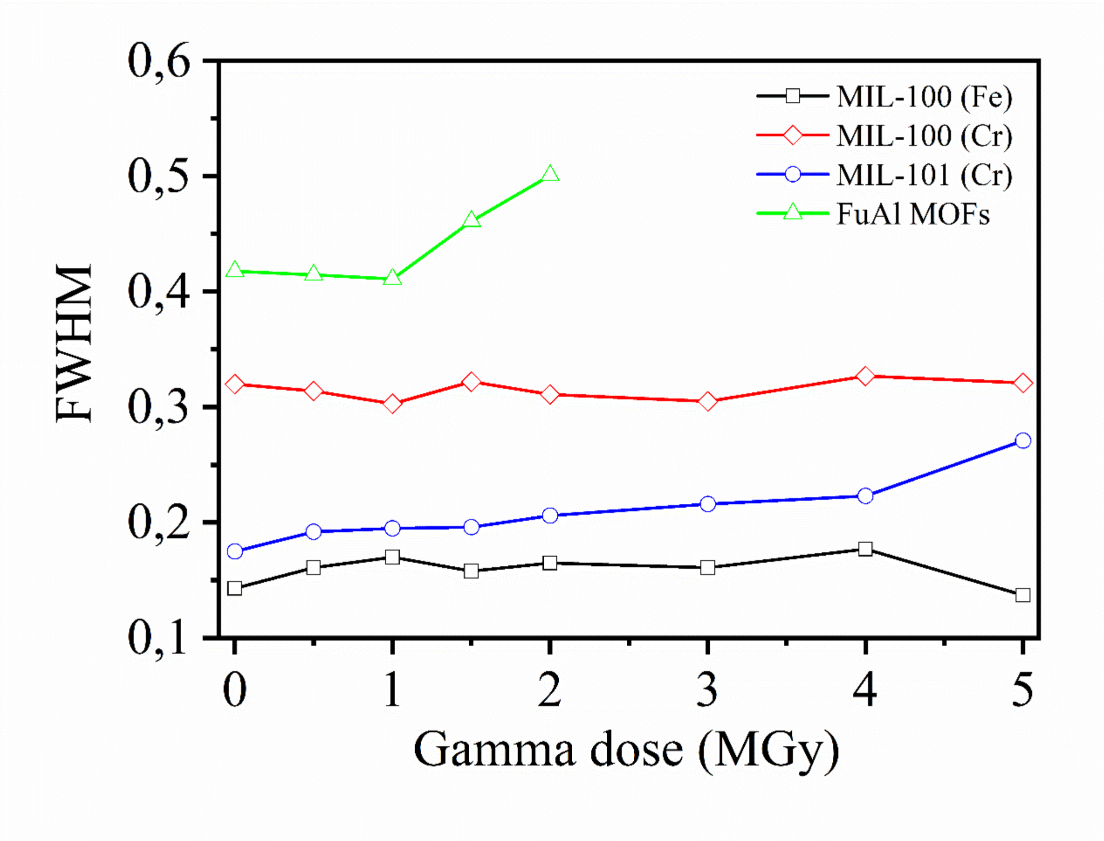

Figure S2. Fluctuations of full width at half maxima (FWHM) for all MOFs under different gamma doses.

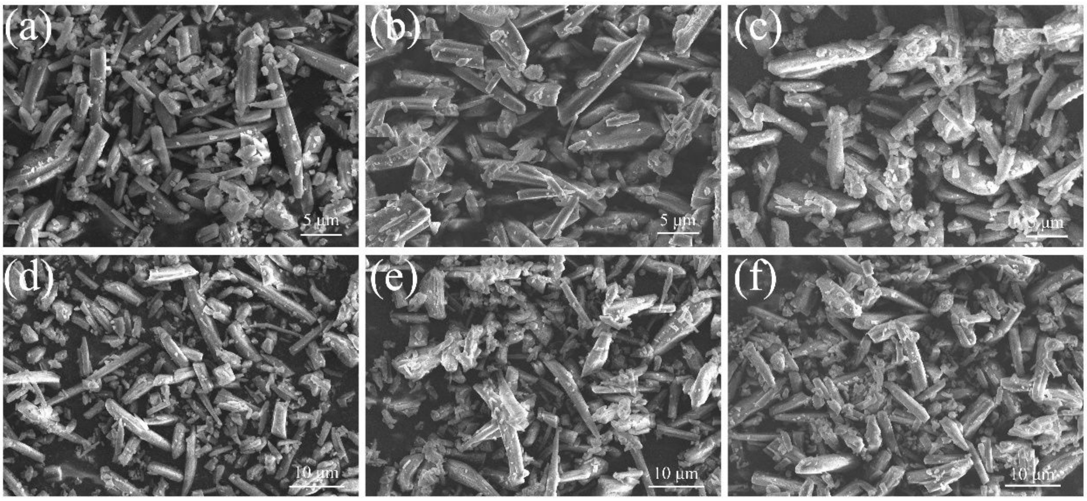

Figure S3. SEM images of MIL-100 (Fe) under different gamma doses: (a) 0 Gy, (b) 1.0 MGy, (c) 2.0 MGy, (d) 3.0 MGy, (e) 4.0 MGy and (f) 5.0MGy.

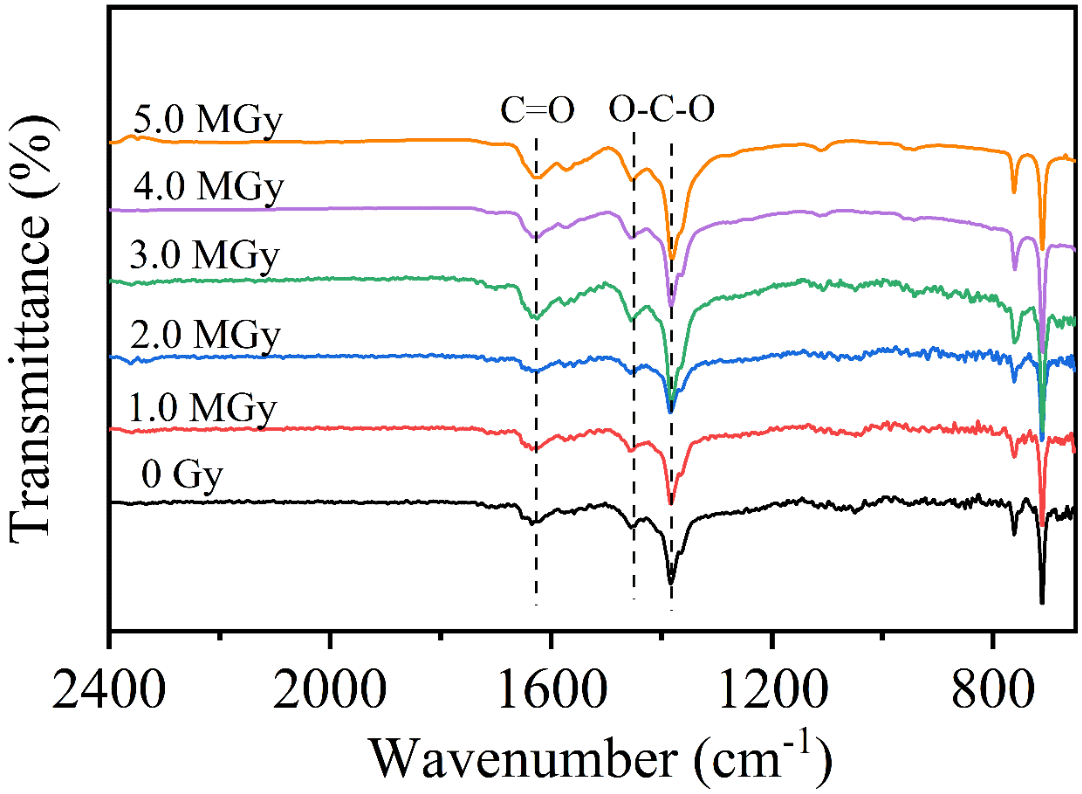

Figure S4. FT-IR spectra of MIL-100 (Fe) under different gamma doses.

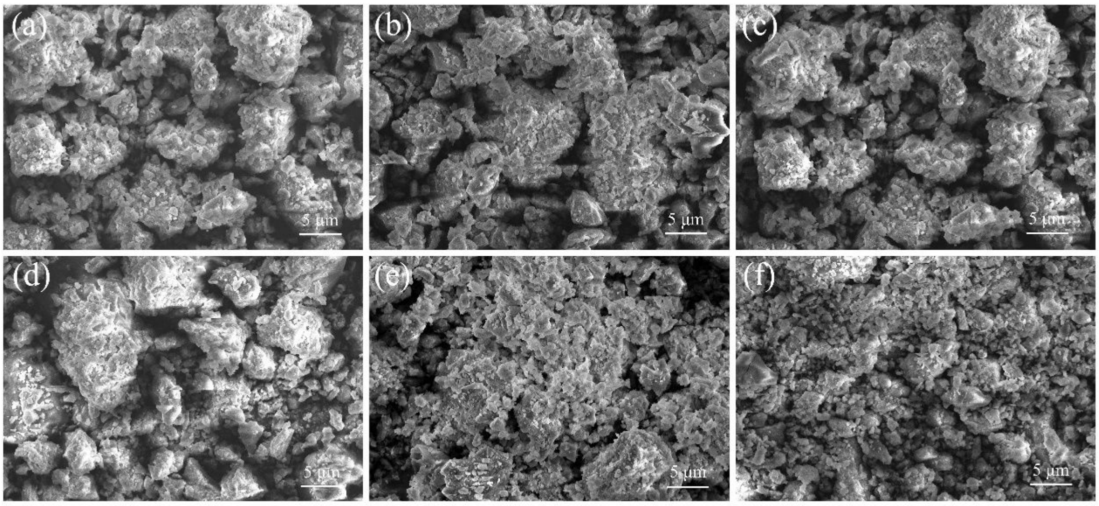

Figure S5. SEM images of MIL-100 (Cr) under different gamma doses: (a) 0 Gy, (b) 1.0 MGy, (c) 2.0 MGy, (d) 3.0 MGy, (e) 4.0 MGy and (f) 5.0MGy.

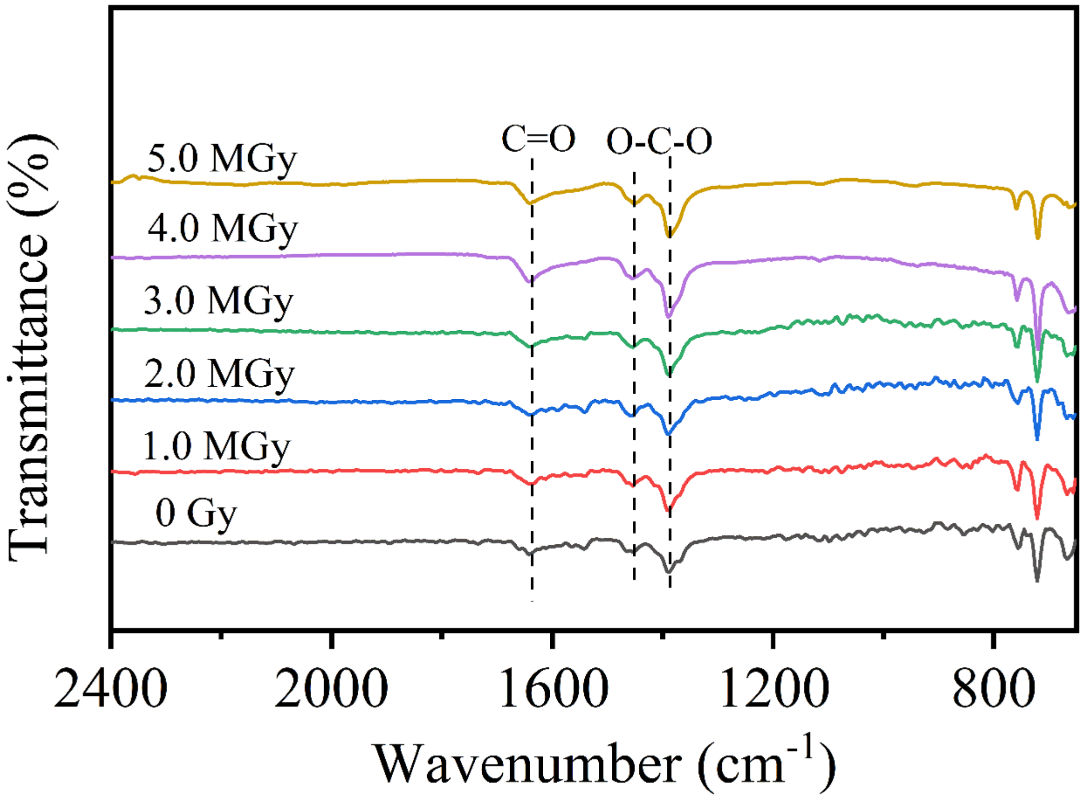

Figure S6. FT-IR spectra of MIL-100 (Cr) under different gamma doses.

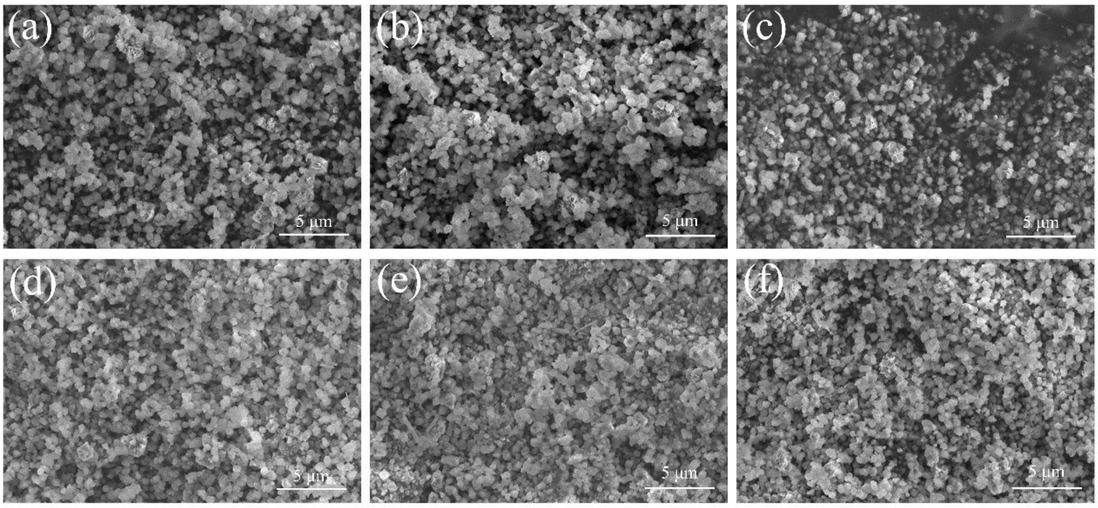

Figure S7. SEM images of MIL-101 (Cr) under different gamma doses: (a) 0 Gy, (b) 1.0 MGy, (c) 2.0 MGy, (d) 3.0 MGy, (e) 4.0 MGy and (f) 5.0MGy.

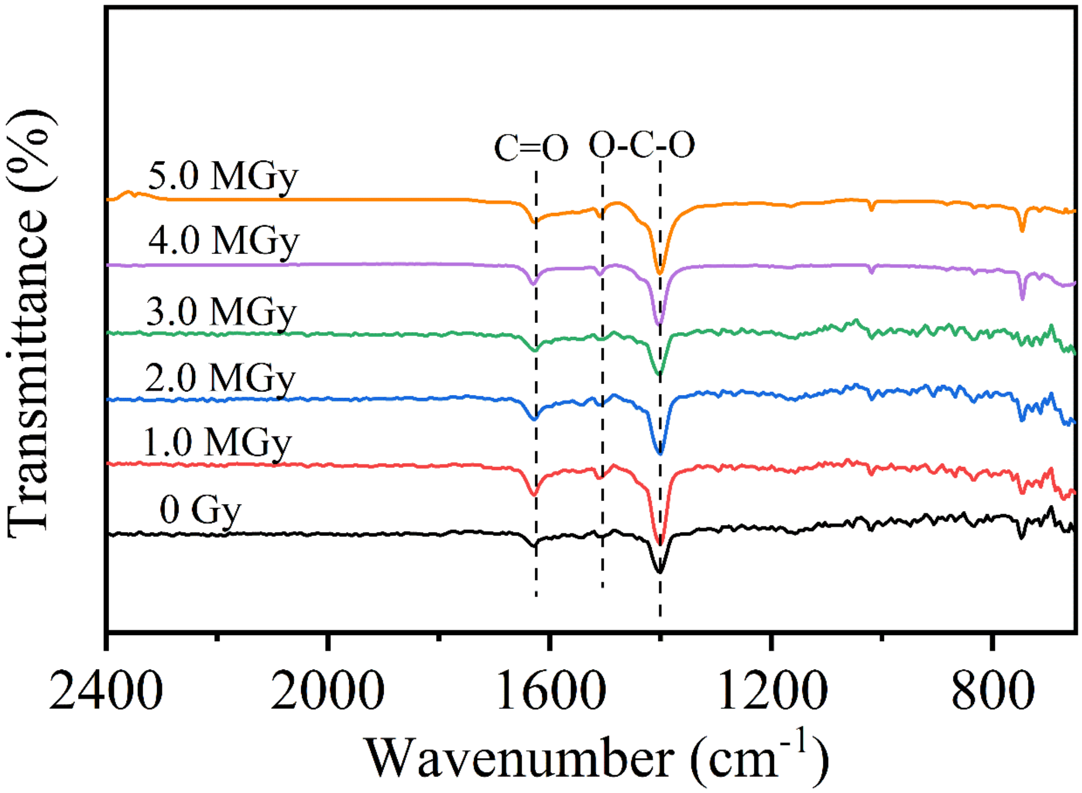

Figure S8. FT-IR spectra of MIL-101 (Cr) under different gamma doses.

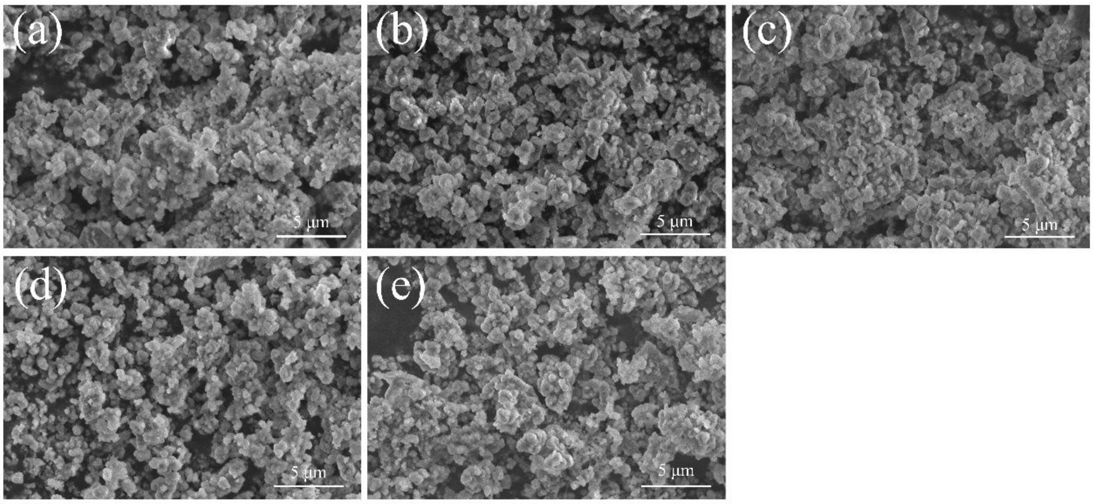

Figure S9. SEM images of AlFu MOFs under different gamma doses: (a) 0 Gy, (b) 0.5 MGy, (c) 1.0 MGy, (d) 1.5 MGy and (e) 2 MGy.

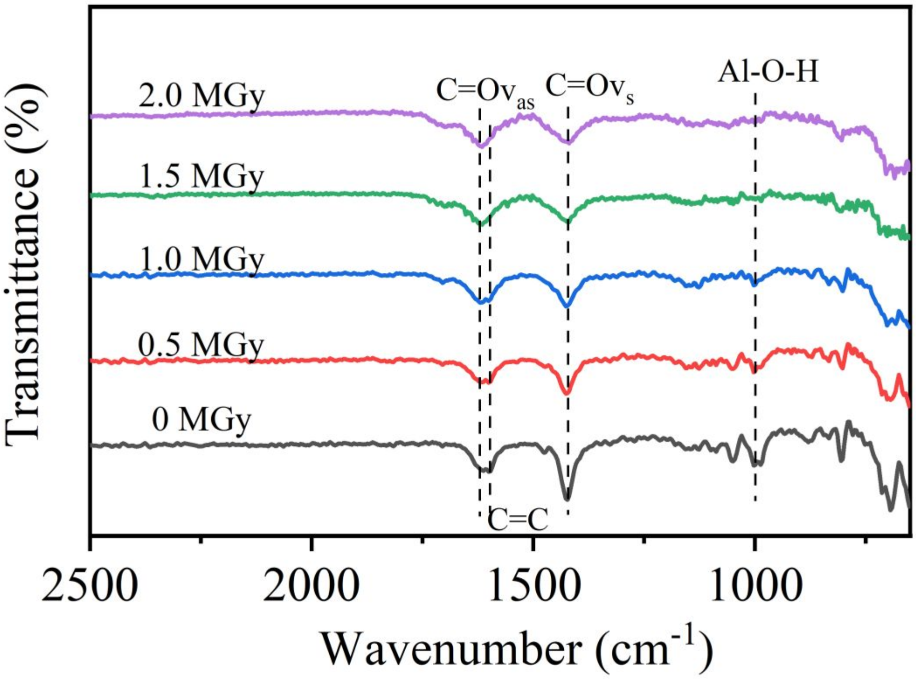

Figure S10. FT-IR spectra of AlFu MOFs under different gamma doses.

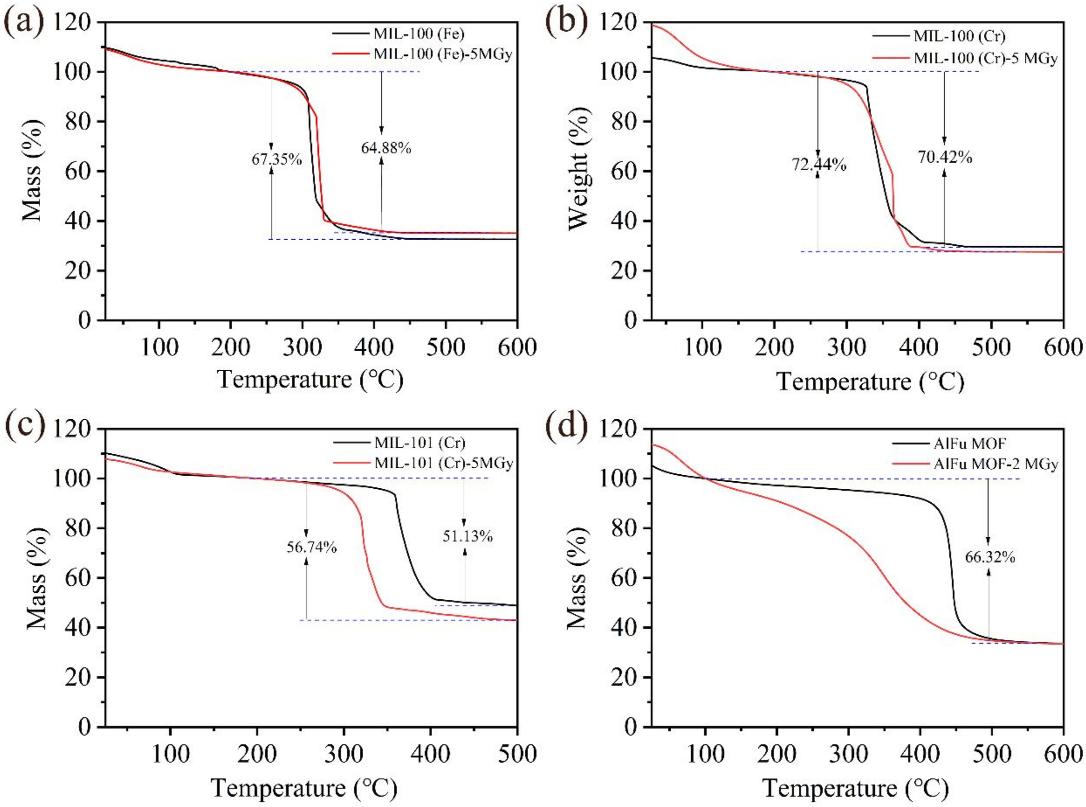

Figure S11. The TGA curves of (a) MIL-100 (Fe), (b) MIL-100 (Cr), (c) MIL-101 (Cr) and (d) AlFu MOFs before and after gamma radiation (5 MGy)

Table S1. Summary of the BET surface area (m^2/g) of the four MOFs before and after gamma irradiation.

Dose (MGy)	MIL-100 (Fe)	MIL-100 (Cr)	MIL-101 (Cr)	AlFu
0	1574±6.6	1862±6.5	2203±13.6	1070±1.3
1	1527±7.0	1802±5.9	2159±25.1	--
2	1528±7.1	1744±5.5	2101±29.8	63±0.1
3	1513±6.5	1660±5.2	2072±28.2	--
4	1498±6.7	1600±5.6	2099±32.3	--
5	1507±6.4	1510±5.9	1762±25.0	--

Table S2. Comparison of the micropore volume (cm^3/g) of the four MOFs before and after gamma irradiation.

Dose (MGy)	MIL-100 (Fe)	MIL-100 (Cr)	MIL-101 (Cr)	AlFu
0	0.6069	0.6699	1.0715	0.3758
1	0.5902	0.6492	1.0331	--
2	0.5894	0.6296	0.9964	0.0077
3	0.5804	0.6097	0.9635	--
4	0.5739	0.5956	0.9372	--
5	0.5775	0.5496	0.7508	--
